# The first 10 years of the international coordination network for standards in systems and synthetic biology (COMBINE)

**DOI:** 10.1515/jib-2020-0005

**Published:** 2020-06-29

**Authors:** Dagmar Waltemath, Martin Golebiewski, Michael L Blinov, Padraig Gleeson, Henning Hermjakob, Michael Hucka, Esther Thea Inau, Sarah M Keating, Matthias König, Olga Krebs, Rahuman S Malik-Sheriff, David Nickerson, Ernst Oberortner, Herbert M Sauro, Falk Schreiber, Lucian Smith, Melanie I Stefan, Ulrike Wittig, Chris J Myers

**Affiliations:** Medical Informatics, University Medicine Greifswald, Greifswald, Germany; Heidelberg Institute for Theoretical Studies (HITS), Heidelberg, Germany; UConn Health, Farmington, CT, USA; Department of Neuroscience, Physiology and Pharmacology, University College London, London, UK; EMBL-EBI, Cambridge, UK; Computing and Mathematical Sciences, California Institute of Technology, Pasadena, CA, USA; University College London, London, UK; Institute for Theoretical Biology, Humboldt-University Berlin, Berlin, Germany; Auckland Bioengineering Institute, University of Auckland, Auckland, New Zealand; U.S. Department of Energy (DOE) Joint Genome Institute (JGI), Lawrence Berkeley National Labs, Berkeley, CA, USA; Department of Bioengineering, University of Washington, Seattle, WA, USA; Department of Computer and Information Science, University of Konstanz, Germany; Faculty of IT, Monash University, Melbourne, VIC, Australia; Centre for Discovery Brain Sciences, The University of Edinburgh, Edinburgh, UK; ZJU-UoE Institute, Zhejiang University, Haining, China; University of Utah, Salt Lake City, UT, USA

**Keywords:** COMBINE, community building, meeting report, standardization

## Abstract

This paper presents a report on outcomes of the 10th Computational Modeling in Biology Network (COMBINE) meeting that was held in Heidelberg, Germany, in July of 2019. The annual event brings together researchers, biocurators and software engineers to present recent results and discuss future work in the area of standards for systems and synthetic biology. The COMBINE initiative coordinates the development of various community standards and formats for computational models in the life sciences. Over the past 10 years, COMBINE has brought together standard communities that have further developed and harmonized their standards for better interoperability of models and data. COMBINE 2019 was co-located with a stakeholder workshop of the European EU-STANDS4PM initiative that aims at harmonized data and model standardization for *in silico* models in the field of personalized medicine, as well as with the FAIRDOM PALs meeting to discuss findable, accessible, interoperable and reusable (FAIR) data sharing. This report briefly describes the work discussed in invited and contributed talks as well as during breakout sessions. It also highlights recent advancements in data, model, and annotation standardization efforts. Finally, this report concludes with some challenges and opportunities that this community will face during the next 10 years.

## 1 Introduction

The COMBINE (http://co.mbine.org/) network (“COmputational Modeling in BIology NEtwork” [1], [2] is a consortium of researchers and software engineers involved in the development of open community standards and formats for computational modeling in systems and synthetic biology (see [3], [4] for recent reviews). The network was formed in 2009 following the observation that many standardization efforts shared similar goals and sometimes even involved the same individuals, and that there were several independent meetings each year for different standardization efforts often involving overlapping groups of people. COMBINE helps foster greater interaction and awareness of the activities in different standards’ communities. This in turn encourages the federated projects to develop standards that are more interoperable and less overlapping compared to non-coordinated development. The COMBINE initiative organizes two annual meetings: HARMONY is a codefest-type meeting that focuses on the hands-on development of standards (with their implementations), as well as on interoperability and infrastructure; the COMBINE meeting is a workshop-style event with oral presentations and discussions, as well as poster and breakout sessions to discuss further directions of the standards’ development. In addition, COMBINE also organizes training events, such as the COMBINE and de.NBI tutorial that has been organized as a satellite of the International Conference on Systems Biology (ICSB) since 2012.

COMBINE 2019 took place from July 15–19 in Heidelberg (Germany), hosted by the Heidelberg Institute for Theoretical Studies (HITS). The 10th anniversary of COMBINE was celebrated with a new record for number and diversity of participants, a new record in number of submissions, and a birthday cake. The following report is both a summary of the meeting and a description of recent developments in the broader COMBINE community.

Over the past 10 years, the COMBINE meeting has become the annual gathering of the standardisation communities in the field. Having been hosted at multiple locations around the world, the meeting remains highly international and interdisciplinary. COMBINE, as an umbrella organisation for standards development in computational biology, coordinates core, associated and candidate standards. Activities are coordinated by the COMBINE Coordination Board, with representatives from all core standards (http://co.mbine.org/about). Core COMBINE standards are BioPAX [5], CellML [6], [7], the Simulation Experiment Markup Language (SED-ML) [8], the Systems Biology Graphical Notation (SBGN) [9], the Systems Biology Markup Language (SBML) [10], the Synthetic Biology Open Language (SBOL) [11], SBOL Visual [12] and NeuroML [13]. Since 2016, the Journal of Integrative Bioinformatics has published annual special issues with updates of COMBINE standards [14], [15], [16].

**Figure 1:**
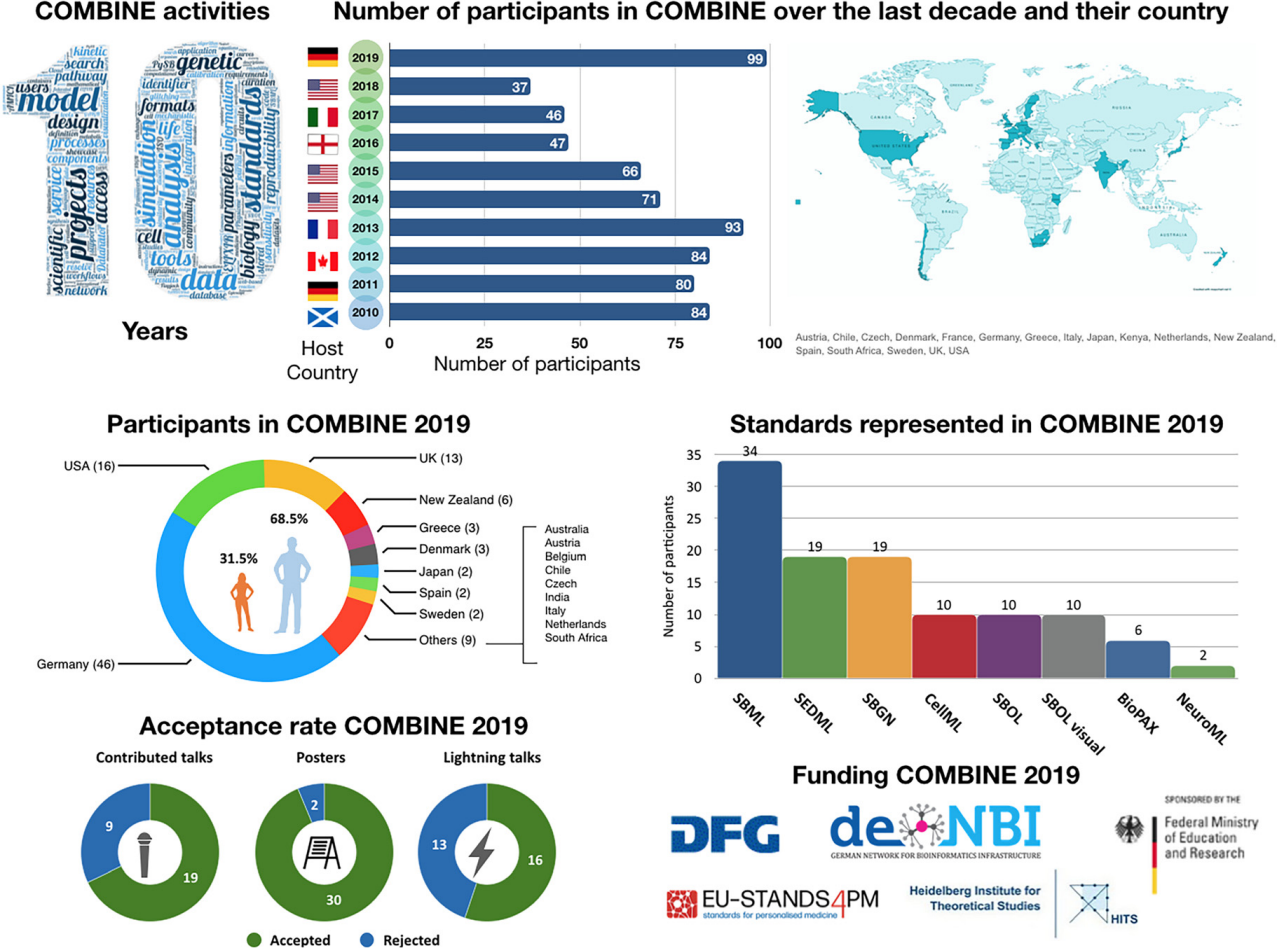
Statistics on attendees of COMBINE 2019.

The 2019 COMBINE meeting had 101 participants from 18 countries and six continents (see Figure 1), including 19 invited and keynote speakers and 19 contributed talks, as well as 16 short lightning talks and 30 poster presentations. Over the past decade, the COMBINE meeting has been held in various locations in Europe and North America with invited speakers, sponsored students, and participants from across the world. The local hosts take turns, and each time sufficient funding needs to be raised to cover meeting costs and travel expenses. COMBINE as a grass-roots standardization initiative has no organization form and thus cannot apply for funding. COMBINE 2019, for example, was only possible due to substantial funding by the German Research Foundation DFG and by the German Federal Ministry of Education and Research through the German Network for Bioinformatics Infrastructure de.NBI, as well as through co-localization of a workshop of the European project EU-STANDS4PM and corresponding financial support by the European Union Horizon 2020 framework programme of the European Commission. To give another example, the US National Science Foundation (NSF) has been a devoted supporter of student travel to meetings on standards for systems and synthetic biology since 2016, funding over 50 students to attend these meetings. Indeed, many of the contributors to standards development are students that volunteer their time and appreciate travel support to discuss their works at the meetings. In 2019 the NSF funding allowed 5 US-based students to travel to the COMBINE meeting. Last but not least, this funding allowed underrepresented groups, including women and minorities, to attend the meeting.

The COMBINE community has always recognized the challenges of travel to its meetings due to the cost, time required, and environmental impact. Indeed, the COMBINE community developed out of a desire to merge several meetings into a single common venue to reduce the amount of travel needed for community standard development. Similarly to all previous COMBINE meetings, COMBINE 2019 involved a number of virtual participants. Due to the ongoing pandemic, the COMBINE Coordinators have decided that COMBINE 2020 will be an all virtual meeting. If this event is successful, we may consider holding one of our two annual meetings virtually every year in the future. While an all virtual meeting presents many challenges, the opportunity to engage a larger community of participants could be a significant benefit.

### 1.1 Co-located events

COMBINE is regularly reaching out to new partner communities. COMBINE 2019, for example, was co-located with a stakeholder workshop of the European EU-STANDS4PM initiative (https://www.eu-stands4pm.eu). EU-STANDS4PM aims at establishing a standardization framework for data integration and data-driven *in silico* models for personalized medicine. A keynote by Carole Goble (University of Manchester, UK) discussed FAIR asset management [3] and introduced tools and services for FAIR [17] data management like the ones offered by the FAIRDOM initiative [18]. Different applications of modeling in clinical practise were presented, such as pediatric oncology. Several “impulse talks” discussed the importance of standards for modelling in personalized medicine. For example, in biobanking it is crucial to trace provenance information about samples. Legal and ethical aspects of modelling in personalized medicine were discussed, and institutions like the Virtual Physiological Human institute (VPHi) were introduced as a means to help to bring the community together and drive *in silico* modelling in medicine. Through the workshop, EU-STANDS4PM has consulted the COMBINE community to put a focus on (i) analyzing interoperability and scalability of data and metadata standards relevant in COMBINE and (ii) reflecting on possibilities for cross-domain and cross-technology data integration to facilitate *in silico* modeling approaches in personalized medicine *(cmp. paper same issue)*.

A second co-located meeting was run by FAIRDOM (https://fair-dom.org/), an initiative to establish sustained data, model and process management service to the European Systems Biology community [18]. With the free SEEK data management software (https://seek4science.org/) FAIRDOM offers a data management platform for interdisciplinary projects to support the storage and exchange of data and models from research partners (https://www.fairdomhub.org) based on the FAIR principles. FAIRDOM PALs (Project Area Liaisons) are “front line” experimentalists, modellers and bioinformaticians from different projects with the intention to build a communication level between users and the FAIRDOM team to collect user requirements, as well as to review ideas and prototypes of novel features in the SEEK software. The PALs also help to test the software in real life, i. e. with original data, metadata and workflows from their own work, and they report back about experiences of their colleagues in the projects with the FAIRDOM data management. For PALs and COMBINE attendees the workshop was a possibility to directly contact the FAIRDOM community and development team, getting personalised advice on using the data and model management platform. Besides presentations by the FAIRDOM team about the current status of the project and future plans, users showed different research projects with advanced data management pipelines. Presentations can be found in FAIRDOMHub (https://fairdomhub.org/events/191).

### 1.2 Colloquium day

The first day of COMBINE 2019 was a colloquium day starting with an overview of the history of COMBINE by Mike Hucka (California Institute of Technology, USA), one of the co-founders of COMBINE. Subsequently, Peter Hunter (University of Auckland, New Zealand) gave a keynote lecture in the HITS colloquium series. He showed recent developments in computational physiology with a focus on novel developments within the Physiome Project [19]. The Physiome Project is developing model and data encoding standards, web accessible databases and open source software for multiscale modeling (http://physiomeproject.org/). In a second keynote Ursula Kummer from University of Heidelberg (Germany) presented “Modeling projects across platforms – the reality and how reality should be” and gave excellent examples about real-life traps, boundaries and unexpected hurdles for modeling.

COMBINE strives to be an open and inclusive community with freely available standards that are developed jointly by the community. This also affects the corresponding publication processes in the domain. In his keynote lecture, Thomas Lemberger (EMBO press, https://www.embo.org/embo-press) shared his experiences and thoughts on implementing open science publishing. Current and planned practises at EMBO press are directed towards an open publishing process. Examples are the open science policies at EMBO press or Biostudies (https://www.ebi.ac.uk/biostudies/) and SourceData services [20]. Open publication of scientific results needs to be paired with better interaction between publishers (of the papers) and data repositories. The current lack of communication between the two players may hinder the publication of reproducible scientific results. The discussion on data deposition led to the question: who should pay for this service? At the same time, the question arose how scientists could be motivated to deposit their data as FAIR data sets?

### 1.3 COMBINE community meeting

The colloquium day was followed by the COMBINE community meeting (Tuesday to Friday). Each day was structured into thematic sessions with selected talks in the mornings and break-out sessions for in-depth discussions in the afternoon. The proceedings of the meeting, including a full agenda and all abstracts, are available online [21] and on the meeting website (http://co.mbine.org/events/COMBINE_2019/agenda).

## 2 Reproducibility in synthetic and systems biology

Several sessions at COMBINE 2019 reflected the continuing interest in further enhancing the reproducibility of simulation studies. The Center for Reproducible Biomedical Modeling (CRBM, https://reproduciblebiomodels.org/), for example, works towards better reproducibility of model-based results in systems biology. In particular, it offers annotation services for composite and harmonised annotations [22], [23], technology development to support the modeling workflow, and training. One major roadblock to automated creation of reproducible simulation studies (and annotations) is the lack of appropriate software tools. Another problem is that the majority of distributed models are only provided as executable code, for example in MATLAB or Python. The question was raised whether the CRBM should focus on a small number of projects to show the utility of annotating models and related data. General consensus at the meeting was that a small number of high quality exemplars was needed to demonstrate the utility of model annotation and to help drive wider adoption.

Further discussions relating to the lack of appropriate software tools showed that the semantic enrichment of models is still tedious, a predominantly manual task that requires time and effort by domain experts, modelers and curators. Data integration, a means to enrich models and link to other knowledge resources, is a key value gained from proper annotation. In addition, annotations increase the level of understanding and the reproducibility of scientific results. When publishing an annotated model in a journal, the quality of the science is increased and the level of frustration is decreased. Not only the quantity but also the quality of annotations is important. The higher the quality and consistency, the more useful semantic annotations become. Measuring the quality of annotations and validation of annotations are two difficult tasks to achieve, as measures highly depend on context and application. Ongoing work on harmonising semantic knowledge about computational models was discussed during the meeting [23], particularly how to realise the next step: going from recommendation to implementation of the OMEX metadata specification.

Another effort to support reproducible modeling, open source software, and multiscale modeling is the *Physiome* journal (https://journal.physiomeproject.org/). It supports different types of publications, amongst them original papers, letters, and retrospectives. The aim is to provide a platform for the publication of reproducible simulation studies, making model code citable, curated, version controlled, and open access. Similarly, the JWS Online platform reports on model validation facilities “on the fly” and using standards for experiment description [24] and exchange (COMBINE Archive) [25].

While a COMBINE Archive bundles all files related to a simulation study in a single package, other options for providing reproducible studies include Docker containers that ship the description of the simulation study together with the actual software and environment that is needed to run the code, or workflows that run the complete analysis pipelines. An approach for implementing reproducible OMICS-profiling pipelines for precision medicine was presented using Galaxy workflows [26] to setup a complex analysis pipeline for profiling tumor biopsy data in late stage cancer. The setup handles large data sets and pathways and it utilizes SBGN diagrams to visualise drug neighborhoods. Reproducibility of simulations that lead to a medical decision, for example on a tumor board, is of particular importance to comply with the regulations for patient safety.

A break-out session on Tuesday discussed specifically how the reproducibility of model-based results can be improved with SED-ML. A particular format had been proposed to enhance the human-readability of SED-ML (XML) – the SED-ML Script. That format follows a script-like procedural semantics, mimics Python syntax and allows access to model elements by treating the model itself as a variable, with its elements accessible as sub-variables. In general, there was enough interest to indicate that further work should be done.

In breakout sessions on Wednesday and Thursday, the SBOL developers also discussed issues related to reproducibility in synthetic biology. In particular, they defined a preliminary list of synthetic biology concepts that can or should be captured in SBOL, including sequence and their annotations, design rules, and provenance. In addition to the requirements of what concepts to capture, the SBOL developers brainstormed on how to bring SBOL closer to experimentalists, such as through re-using and developing novel tools and features. Finally, to lower the entry bar of SBOL, the SBOL developers brainstormed about ways for the community to better share experiences with methods and software for data sharing.

## 3 FAIR Managers – handling evolving, distributed data and models

The FAIR principles apply to COMBINE, and much of the ongoing movement is relevant to the work relating to reusability and reproducibility of models. Bringing FAIR to reality, however, poses many challenges. Unfortunately, it is quite difficult to make FAIR more defined and actionable. Ongoing efforts aim to measure FAIRness, and there is now a jungle of projects and initiatives that take on the tasks of making data FAIR by defining metadata standards and minimum information for data exchange. These efforts should ideally not only focus on repositories as the “last mile” but also on the source of data production at the “first mile”. Another practical difficulty is the connection of distributed datasets that may be based on common standards. The ELIXIR Cloud and Authentication & Authorization Infrastructure (AAI) project establishes a network between ELIXIR nodes for Human Data Communities that comply with the Global Alliance for Genomic Health standards and specifications (GA4GH) [27]. It delivers the platform for large-scale integration and standardisation of genomic and phenotypic data as well as sensitive human data.

Another example for data accessibility is the recently announced *BioModels Parameters* resource. It allows the search and access to parameters values extracted from about 700 curated models stored in the BioModels database [28] (https://www.ebi.ac.uk/biomodels/parameterSearch/). The data entries are cross-referenced with resources like UniProt [29], Reactome [30] or SABIO-RK [31] to increase interconnectivity.

It was also discussed how the domain-specific and cross-domain harmonization of standards developed by the scientific community (e. g., by COMBINE and other initiatives) and standards defined by more official standardization bodies (such as the International Standardization Organization ISO or national bodies like the German DIN) together may support the integration of complex and heterogeneous data and models, following different formatting and metadata standards. If these domain-specific standards are made interoperable, the data formatted and described according to those standards becomes interoperable and consequently integratable. In particular, the broad positioned standardization bodies (ISO, DIN, etc.) with their domain-overarching pool of experts drafting the standards might help to harmonize domain-specific standards from the scientific communities also across the domains, and with their resources provide a platform for long-term sustainability. For instance, the ongoing definition of novel ISO standards in the life sciences by standardization committees such as ISO/TC 276 Biotechnology (e. g., the emerging standard ISO 20691) or ISO/TC 215 Health Informatics may help to define a domain-overarching framework and guidelines for community standards and their applications.

Discussion at the *inaugural meeting of the ModeleXchange consortium*, initiated by Henning Hermjakob, stressed that repositories play an essential role in making models easily shareable and accessible. A common platform that will allow scientists to search for models across all existing repositories is highly desirable. This requires model repositories to share the model metadata in a common platform. A ModeleXchange consortium could collaboratively develop such a platform and facilitate the development of common and shared curation standards and pipelines as well as provide opportunities for repositories to support each other. The ideas on ModeleXchange are based on the highly successful ProteomeXchange [32], [33] and IMEx [34] collaborations in mass spectrometry and interactomics, respectively. The idea of forming such a consortium led to lively discussions, the majority of people who spoke up were in favor of bundling resources. People even went further, suggesting a COMBINEArchiveXchange resource, which would allow for annotated COMBINE Archives (instead of models) with detailed version information on the set of files relating to a simulation study. The provision of some quality assessment level was discussed, and the question was raised how additional data would fit into the resource? In relation to the earlier discussion about open publication, it was pointed out that ModeleXchange may also foster communication and push collaborations.

## 4 COMBINE standards in the wild – biomedical applications and emerging needs

The meeting collected different applications of modeling and simulation on medical science. For example, personalized modeling pipelines for cardiac electrophysiology simulations are used to predict cardiac resynchronization therapy in infarct patients [35]. To model the heart, multi-scale approaches are required ranging from electrophysiologcial models of single cells (describing activation and repolarization), to tissue scale models based on homogenization approaches which are scaled to the complete organ. For efficient computation, model reduction approaches are needed. The resulting model allows the systematic study of the effect of pacemaker location on repolarization. A key result of the model was that lead location near the structural heterogeneous tissue (scar) resulted in increased repolarization heterogeneity and ventricular arrhythmia. Challenges include further validation and increasing patient recruitment numbers.

Different modeling approaches, from ODE-based to logic and statistical models, and analysis tools are already covered by COMBINE standards. For example, COMBINE standards contribute to automating the modeling and simulation pipeline for liver function tests (https://livermetabolism.com): physiological based pharmacokinetic models can be encoded using SBML Level 3 [36] in combination with hierarchical model composition (SBML comp) [37]; and model simulations can be encoded using SED-ML [38]. While COMBINE standards have advanced and can map most of the modeling and simulation tasks, the acquisition of high-quality data was identified as a current bottleneck. A crucial building block for computational models is the availability of high quality curated and annotated datasets. As part of the project PK-DB such a database for pharmacokinetics data has been established [39]. However, manual extraction and curation of biomedical data for clinical applications does not scale. A vision for the near future is thus to run stratified and personalized liver function tests based on clinical data through a simple app in real-time.

The NIH SPARC project (https://commonfund.nih.gov/sparc) evaluates the use of functional descriptions of organ anatomy to generate annotated 3-dimensional geometric models for a range of species being performed as part of the model was presented. This work particularly emphasized how the adoption of harmonized annotation approached across COMBINE will enable the integration of the wide range of models and data.

Certainly, agent-based modeling offers new possibilities for modeling of biological systems: agent-based modeling is centered around the system’s entities, state transitions are defined by functions and the system can be represented in great detail. However, a lack of standard-compliant methods and tools restricts the practical work of modelers. While the ODE-based modeling world offers sophisticated software tools such as COPASI [40] or RoadRunner [41], usability of agent-based models and the reproducibility of the results strongly depend on the capacities and willingness of the individual modeler. A few software tools already provide tool-specific formats and methods, including MORPHEUS [42], Swarm (http://www.swarm.org/), NetLogo [43], and openABM (https://www.openabm.org/), but these are not interoperable between tools. A discussion is necessary of how to engage with the community on how to form their own standards. Whenever possible, new developments should reuse existing COMBINE formats and infrastructure and comply with the community guidelines.

## 5 Software development

Kinetic modeling in the form of ordinary differential equation (ODE) systems is one of the central methods in computational biology. A crucial step is the calibration of these models based on experimental data. Whereas finite differences and forward sensitivities scale linearly with model size (parameter number), the adjoint sensitivities scale constant allowing to calibrate very large models [44]. These gradient methods have been implemented in another tool presented at the meeting, AMICI (https://github.com/ICB-DCM/AMICI), an advanced multi-language interface to CVODES and IDAS implemented in C++ with language bindings for MATLAB and Python. Applications range from rule based models of resistance in melanoma to automatically assembled models of cancer signalling [45], [46].

*The Datanator* (http://www.datanator.info) is a software tool for discovering, aggregating, and integrating data for whole-cell modeling [47]. Data from various sources are required for the construction of such models ranging from metabolite data (ECMDB) [48], over kinetic parameters (SABIO-RK) [31] to enzyme amounts (paxdb) [49]. Data is hard to utilize for modeling because data is scattered across a large number of sources, and described with inconsistent identifiers, units, and data models. This makes data aggregation a main challenge for large-scale modeling [49], [50]. Future work will focus on incorporating additional data sources, expanding the frontend and allowing additional groups to integrate data, making the Datanator a community resource.

The latest updates were also presented on OpenCOR (https://opencor.ws/), an open source cross-platform modeling environment for organising, editing, simulating and analyzing CellML-encoded models. The plugin-based application integrates tightly with the Physiome repository (PMR) [51] allowing to work with workspaces in the repository. This allows cloning model workspaces and track changes (similar to Git). OpenCOR provides a python interface which allows use for instance in Jupyter Notebooks. Models can be translated to multiple target languages, for example, C, Fortran 77, MATLAB or Python. The future plans of OpenCOR include full Python support and Python based plugins, finalization of the plotting of external data, usage of external data to drive models, move gradually to CellML 2.0 and implement additional features of SED-ML L1V4.

VCell is a virtual cell modeling and simulation tool [52] that supports various mathematical frameworks (such as ODE, stochastics, PDE, rule-based modeling, and particle-based spatial stochastics) and enables distributed simulations (cluster-based and client-based). An approach for precise and compact representation of rule-based models is implemented in VCell [53]; it is based on the three basic concepts that allow scalability: molecular pattern (molecules that participate or are affected by the rule), rule center (molecular sites that are directly modified by a rule), and rule context (molecular sites that affect the rule).

Repeatedly, the COMBINE community discussed a lack of appropriate software tools and shortcomings with the implementation of standards in the tools available. The community observes a discrepancy between the ideal implementation of cross-platform modeling projects and current reality. A recurring problem is the inability to secure funding for tool development – updates in COMBINE standards need to be reflected in the software libraries and then be incorporated into the software tools supporting the standard. This work is rarely funded explicitly, but requires community engagement. For example, while many software tools for pathway modeling support SBML and SBGN [54], some of them may only support part of the standard. When funding for a research project ends, the software development often stalls, meaning that the software tool does not support the latest version of a standard after a short while. Research software is then not maintained without funding, making it more difficult for users to make a choice for the most suitable tool. This situation could be improved with the implementation of Research Software Engineers (RSE) in the academic world, preferably in permanent positions. This certainly requires rethinking in universities and research institutes, but first evaluations show that researchers indeed request help from RSEs and that they are willing to pay for the service [55]. Further support is provided through initiatives such as the Software Sustainability Institute in the UK [56] and the German organisation for Research Software Engineering (de.RSE, https://www.de-rse.org/). In addition, the community seeks to secure funding for sustainable software development – a topic that is also discussed during the regular COMBINE coordinator’s meetings and taken seriously by the chair and vice-chair of COMBINE.

## 6 Model semantics and annotation

Metadata describing the semantics of models and their constituent parts are indispensable for understanding and reuse of computational models. In 2005, the community agreed on the MIRIAM guidelines for good practices in model annotation [57]. Because the original guidelines do not sufficiently cover all aspects for modern model annotation, “MIRIAM 2” has been informally discussed. In addition, the OMEX Metadata Specification 1.0 has now been drafted *(OMEX specification, same issue)*. The OMEX format describes the content of the abovementioned COMBINE archive. It is a technical document describing how to encode semantic knowledge about models, and how to link this knowledge to model parts. A central question during the COMBINE meeting indeed was how the community can make annotations work properly. Several initiatives beyond COMBINE struggle to find standards for data annotation. In models, composite annotations [20] an be very complex and thus be difficult to generate and interpret without computational support. A central repository of such annotations would speed up the annotation process, allow modelers to retrieve sets of annotations for model elements, and – together with a sophisticated user interface – enable more researchers to add annotations to their models easily. Developments in this direction are a library for semantic annotations (https://sys-bio.github.io/libsemsim-docs/) and the abovementioned ModeleXchange consortium. A common library for annotations would also harmonise semantic representation across domains, on both the technical level and with respect to the ontologies and terminologies used. In practise, however, model annotations are diverse. For example, in genome-scale modeling, it is common to merge models into a single representation, and such models can end up with very mixed sets of annotations (quality-wise and with respect to the resources used for annotation). To date, the community is not aware of a software tool that is capable of harmonising annotations.

Another interesting question is how to design software that can complete missing annotations automatically. Arguably, a number of ontology browsing tools [58], [59] and software for model annotation are available [58], [60], [61]. However, annotation tools are not being used at large scale. One reason could be that semi-automatic approaches do not scale to large models. Adding annotations to the models afterwards, however, is a difficult task. The discussion of suitable tools for model annotation was extended to data annotation, a process that is also hard to handle at the moment. While there are already recommendations, for example by the DataCite project [62], or the Open Knowledge Foundation using JSON [63], these are not used in the COMBINE community. A solution could be to extend the OMEX specification to also cover data annotation. In addition to COMBINE-wide efforts to harmonise annotations, model curators like the BioModels team have their own internal curation procedures, which includes a list of preferred annotations, for example, using ChEBI [64] for chemicals. Similarly, the Center for Reproducible Biomedical Modeling has collected a set of models comprehensively annotated with biological semantics following the SemGen annotation protocol (https://reproduciblebiomodels.org/gold-standard-models/). After these examples, it became clear that – again – a stronger link between tool developers, curators and the COMBINE community is necessary, exchanging experiences, best practices, and software code. While the community did not agree to recommend certain ontologies, it was considered a good idea to build a set of gold standard models. Suggestions for candidate models to augment the current set introduced above should be requested from the different modeling communities. In addition, annotation jamborees were mentioned as a way to improve current practices and grow the set of gold standard annotated models. No conclusion was reached for the question whether the COMBINE community should in the future make recommendations for which ontologies to use in annotations, including a ranking from ontologies that were “good to use” to ontologies that were only for “emergency use”.

As the availability of richly annotated models grows, interesting applications emerge, for example, novel model discovery and composition [65] and the linking of computational models to health data. Additionally extending annotation methods to new types of semantic knowledge and new methods to describe the knowledge are actively being pursued. For example, the Gene Ontology Causal Activity Modeling (GO-CAM) will enable new applications of GO in pathway and network analysis [66].

## 7 What’s new? Progress in standard development

The SBML Level 3 Core specification has been stable and well supported for some time. Ongoing work focuses on the Level 3 packages as optional extensions to the core specification. The Flux Balance Constraints (“fbc”) package [67] has additions that have been agreed on for Version 3. Implementation of the new features in various tools is the next step, for example in CBMPy [68]. Updated MATLAB bindings for libSBML [69] will be required for support in COBRA Toolbox [70]. Two other SBML packages are nearing completion: the Distributions package (“distrib”) and the Spatial Processes package (“spatial”). With regard to implementation, Antimony [71] and sbmlutils [60] support the *Uncertainty* element defined by SBML Distributions package. COPASI [40] and iBioSim [72] support representing distributions as an annotation inside SBML files and could use the converters provided by libSBML to read the new, officially-approved format. libRoadrunner [41] is working on supporting distrib. The “spatial” package has a number of smaller technical issues that need to be resolved. It was concluded that the specification needs some alteration and additional explanations. Despite being in draft state, the specification is already supported by CellDesigner [73] and CellOrganiser. VCell [53] supports a previous version of the specification.

To represent multi-cellular models in SBML, the Level 3 Arrays package and the Dynamic Processes package would both be essential, but no one is currently driving their development. However, the new MultiCellML (https://multicellml.org/) effort will provide support for these types of models. Its logic follows the SBML structure, rendering this effort compatible with SBML, maybe even as a novel SBML package in the future. Solutions found may be portable back to SBML if this effort bases the encoding on SBML as far as possible. All agreed that this would be a sensible approach.

Multi-cell models in computational neuroscience are represented using NeuroML. NeuroML’s capabilities range from specification of single cells to complex 3D neuronal circuits. The Open Source Brain (OSB) Initiative runs a structured database of well-tested and curated NeuroML models [74]. OSB seeks to encourage open collaboration to develop the models, and the code for the models reside on GitHub, with access control (e. g. deciding who gets write access) staying completely with the model authors. OSB also offers a web-interface for visualising, analyzing and simulating the NeuroML models.

The Systems Biology Graphical Notation (SBGN) focuses on the graphical representation of biochemical processes and biological networks. New features of Process Description (PD) language Level 1 Version 2.0 [75] simplify the language and include new glyphs for equivalence operator, annotation, submap terminal, empty set replacing source and sink, and subunits. Several updates were presented for tools that either use SBGN for visualization (CellDesigner, Pathway Commons [76]) or plan to use SBGN (VCell, PySB, SynBioHub). The accompanying SBGN workshop provided a platform for intensive discussions on the SBGN future. It addressed questions of (i) how to join SBGN maps of different languages, (ii) how to make maps dynamic and (iii) how to display annotations. Merging SBGN diagrams may require combining or joining different SBGN languages (PD, ER, AF) in one diagram. For example, SBGN Activity Flow (AF) [77] may impact the PD reactions as given, for example, by gene-protein-reaction rules in constraint-based modeling. Pros and cons of different approaches have been discussed, but no conclusion has been reached. Dynamic SBGN will enable animation of maps and showing changes over time. A relevant issue is the display of submaps and annotations. The community started a discussion if visualization approaches (dynamical visualization, submaps, annotations) should be part of the SBGN languages or only of SBGN-ML [78], or if a manifest file should be introduced. However, all these issues will require tool support and thus were raised but not resolved pending the prototype implementation in visualization tools. Follow-up discussions will take place during next meetings.

Also the study of complex multicellular systems requires specific tools to store, analyse and visualise experimental data. One example is FlapJack (https://github.com/SynBioUC/flapjack/), a docker-based web service for storing, visualising and analyzing gene expression data. It facilitates the integration of models and experiments in synthetic biology. FlapJack integrates several software tools such as SynbioHub [79] and SBOLDesigner [80].

The first session on Tuesday focused on standards for Synthetic Biology (https://sbolstandard.org) and their applicability in synthetic biology design-build-test-learn (DBTL) workflows. Recently, the “SBOL Industrial Consortium” (https://sbolstandard.org/sbol-industrial/) was created with the goals (i) to enhance the adoption of the SBOL standard in industry and (ii) to incorporate industry requirements into the development of the standard. SBOL’s approach of tackling the problems of reproducibility of synthetic biology workflows and results is to track the provenance of entities (e. g. sequences, experimental results). To this end, the SBOL developers recently integrated the W3C Provenance Ontology (PROV-O) (https://www.w3.org/TR/prov-o/) into the SBOL data model, enabling them to capture provenance information across an entire DBTL cycle using SBOL and its adopted PROV-O concepts. The SBOL community is currently working on SBOL Version 3 *(cmp. Publication in the same issue)*, which will substantially simplify the representation of genetic design information. SBOL is utilized in multiple software tools. In particular, the Design, Implementation, and Validation Automation (DIVA) workflow includes the software tools j5 (https://pubs.acs.org/doi/abs/10.1021/sb2000116), openVectorEditor (https://j5.jbei.org/VectorEditor/VectorEditor.html), and the Build-Optimization Software Tools (BOOST, https://pubs.acs.org/doi/abs/10.1021/acssynbio.6b00200). Another example for a workflow featured the integration of SBOLDesigner [80] and BOOST. Finally, a fully integrated computer-aided design (CAD) workflow for the design of gene cluster refactorings substantiated the value of SBOL and its provenance tracking capabilities. However, the challenge of provenance visualization tools remains.

SBOL can be connected with other standards, for example SBML, via dynamic model generation procedures. This procedure begins with a genetic design represented in SBOL, fetches genetic part characterization data from the Cello project [81] stored in a SynBioHub data repository [79], and creates a ODE model represented in SBML for simulation with the iBioSim software [72], clearly demonstrating the value of the interoperability of COMBINE data standards. Another major international standardization effort in synthetic biology is BioRoboost (http://standardsinsynbio.eu/), a project funded through the European Commission H2020 Research & Innovation Programme. Manuel Porcar presented the challenges of reproducibility in synthetic biology, as well as standardization and bio-security, based on the International Genetically Engineered Machine (iGEM, https://igem.org/) competition.

## 8 Emerging standardization needs and multicellular modeling

With the emergence of multi-scale models comes the need for new standards and procedures. Multi-scale models, for example, encode the cellular and molecular processes underlying learning of the vestibulo-ocular reflex, accounting for both electrophysiological and biochemical events in medial vestibular neurons. This requires combination of two models: one of electrical signalling within neurons of the medial vestibular nucleus [82] from ModelDB [83] and one SBML model of postsynaptic chemical pathways [84] from BioModels Database. The combined model offered new insights into an unusual form of synaptic plasticity and is a great example of model re-use from different public resources, but also raises questions of how to standardise and document decisions made when writing code to bridge models across scales. Amongst the suggested improvements were meaningful annotations of model components, detailed description of model interfaces (e. g., using port definitions in SBML comp); and definition of units for all model components. Specifically the latter is a prerequisite for model coupling and scaling of model outputs across scales (e. g., to calculate conversion factors between model components).

The FindSim platform [85] facilitates collaboration between modellers and experimentalists on a project investigating neuronal signalling in Autism Spectrum Disorders. FindSim makes use of SBML to describe the model, but found it challenging to integrate other standards, especially when it came to capturing experimental data and comparing model outcomes to experiments. A possible solution to these problems is the use of SED-ML.

For the representation of complex and dynamic models of cells, the main challenge is that there is currently no language to capture the complexity: Different frameworks exist for specialised purposes, but it is not clear how they relate to each other. One solution may be a unified model description language (MDL) based on biophysical notation, independent of the modeling framework, modular with support for standards, and extensible. It should also support nesting of spatial components, for instance, it should be possible to model a nucleus nested within a cell that would inherit properties from its parent cell. The Morpheus modeling and simulation environment [42] provides such a model description language.

## 9 Conclusion & future work

The COMBINE community, founded 10 years ago, used the 2019 anniversary to look back at its achievements and to plan ahead. The meeting attracted users from diverse modeling communities, specifically from the clinical and biomedical domains. As a result, COMBINE 2019 not only provided an update on standards and software tools, it also launched discussions on cross-standard topics including reproducibility, open science publications, science communication, and research software engineering. The meeting tried new formats for session organisation and communication. World cafes and lightning talks made the meeting more lively and more interactive.

The co-location with the EU-STANDS4PM workshop reflects the move of computational biology towards medical applications. The COMBINE community will need to open up to the specific requirements and regulations that apply when bringing models into the clinic for diagnostic and therapeutic use, and experts will be needed in COMBINE to direct these developments. At the same time, this also shows that COMBINE should follow the path further to strategically collaborate with other networks and initiatives, as well with regulatory and normative bodies.

The COMBINE community provided solutions to the computational modeling community in the last decade, among them the standard formats for model representation (SBML, CellML, NeuroML), description of simulation experiments (SED-ML), visual representation of models (SBGN) or representation of information in synthetic biology (SBOL, SBOLv). Many technological, but especially social and cultural challenges lie ahead. One remains the outreach to researchers, software developers, and funders, promoting the use and further support for COMBINE standards. A major point is the continuation of training, especially to students and early-career scientists. We desire a closer interaction and more frequent communication with scientific journals and reviewers of model-based publications. Another goal is that model curation becomes part of the standard modeling and review process.

COMBINE is a community-based effort that requires active and regular feedback from its members. A world cafe at this year’s COMBINE Forum aimed to share experiences in building a standards community. Around 20 participants discussed in three different groups what aspects of COMBINE work well, why COMBINE had been successful in implementing standards across scientific communities, how COMBINE could become more visible and spread further, and how COMBINE could benefit from the adoption of efforts from other communities.

What are the plans for the future – for COMBINE as a community? That question had been discussed both amongst the participants and amongst the COMBINE coordination board. An intermediate result was the formation of a chair – and vice-chair position for that board. These new positions are direct contact persons for other standardisation bodies, funding bodies, journals, and for the community. Furthermore additional measures for transparency have been defined and meanwhile been implemented by the COMBINE coordinators, for example the publication of meeting notes from the coordination board (http://co.mbine.org/documents).

**Figure 2:**
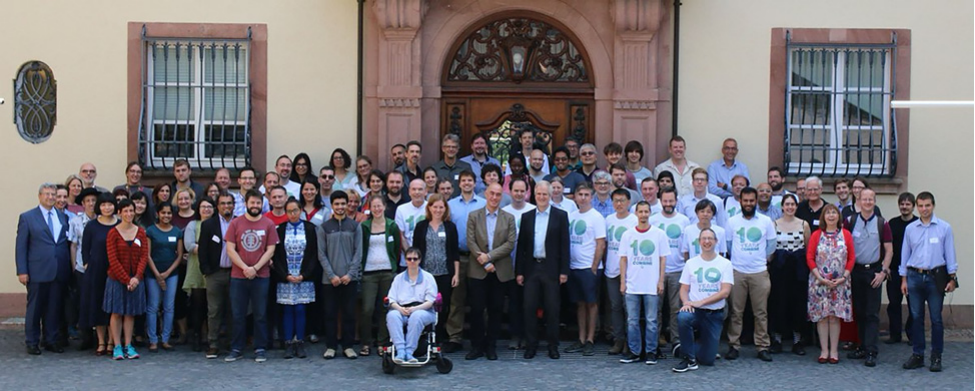
Participants of the 2019 COMBINE meeting.

Last but not least we would like to thank all attendees of this year’s meeting for their valuable input and contributions (Figure 2).
